# Longitudinal Computed Tomography Indicates No Negative Impact of OnabotulinumtoxinA on Mandibular Bone Density in a 12-Month, Double-Blind, Randomized, Repeat Treatment, Placebo-Controlled Study in Healthy Adults With Masseter Muscle Prominence

**DOI:** 10.1093/asj/sjaf167

**Published:** 2025-08-22

**Authors:** Paul Kostenuik, Sanjay M Mallya, René Hopfinger, Tara Aghaloo, Shawneen Gonzalez, James Mah, Mansur Ahmad, Grace Pan, Donna L Faletto, Elisabeth Lee, Beta Bowen, Mitchell F Brin

## Abstract

**Background:**

Botulinum neurotoxins are used to treat masseter muscle prominence (MMP), benign bilateral masseter muscle enlargement which can be aesthetically undesirable. Limited data report botulinum neurotoxin injections in the muscles of mastication, with subsequent reduction in biomechanical loading, may impact mandibular bone density.

**Objectives:**

This study evaluates whether changes in mandibular bone density occur after bilateral masseter treatment with a botulinum neurotoxin, onabotulinumtoxinA, in individuals with MMP.

**Methods:**

Analyses were performed on a prespecified subpopulation (*n* = 123) from a 12-month, double-blind, placebo-controlled, dose-escalation, Phase 2 study (*N* = 187). Participants received 1 or 2 bilateral masseter treatments of onabotulinumtoxinA (48, 72, or 96 U) or placebo and had multi-detector computed tomography scans at baseline and Days 90 and 360 post-treatment. Cortical and trabecular bone densities, estimated in Hounsfield units, were calculated for the bilateral condyle, premolar dentoalveolus, and ramus.

**Results:**

No clinically significant changes in mandibular bone density were observed in condyle, premolar area, or ramus of onabotulinumtoxinA-treated participants, when compared with placebo or baseline, after 1 or 2 treatments.

**Conclusions:**

In healthy adults with MMP, 1 or 2 bilateral masseter treatments with onabotulinumtoxinA at doses of 48, 72, or 96 U over 1 year did not negatively impact mandibular bone density.

**Level of Evidence: 1 (Therapeutic):**

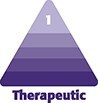

The masseter muscle is 1 of 4 bilateral muscles of mastication. Benign bilateral masseter enlargement, or masseter muscle prominence (MMP), appears as a wide or square lower face shape that some find aesthetically bothersome.^1-4^ Historically, cosmetic surgery was performed to resect a portion of a hypertrophic masseter muscle.^5,6^ Over the past 30 years, botulinum neurotoxins, including onabotulinumtoxinA (onabotA; Botox Cosmetic; Allergan Aesthetics, an AbbVie Company [Irvine, CA]), have been used as a non-surgical treatment for MMP.^7,8^ OnabotA injections in the masseter reduce local muscle activity, decreasing muscle volume and producing a narrower, more ovoid lower facial appearance.^9,10^

Preclinical studies report botulinum neurotoxin type A (BoNT/A) injections in the masseter muscle of rabbits and rodents, often in combination with the temporalis, may alter mandibular bone morphology, thickness, and density.^11^ The clinical relevance of these preclinical studies to humans is unclear, owing to inter-species variations in mandibular bone anatomy, physiology, and relative reliance on masseters for mouth closure.^12-16^ Moreover, most of the preclinical models used skeletally immature rodents, limiting translation of these findings to clinical treatment of healthy adults with MMP.^17^

Clinical reports of mandibular bone changes following BoNT/A treatment have primarily involved adults with confounding morbidities (eg, temporomandibular joint disorders [TMDs], myofascial pain syndrome), who received injections into multiple masticatory muscles (eg, temporalis, pterygoid) rather than masseters alone.^18-22^ These distinctions matter, because, for example, TMDs have been linked to parafunctional masticatory activity and osteoarthritis.^23^ Furthermore, studies have employed cone-beam computed tomography (CBCT) or other CT scans to evaluate fine structures of the mandible, pushing these technologies to their resolution limit.^24,25^

Two recent systematic reviews examining effects of BoNT/A treatment of masticatory muscles on mandibular bone concluded a low quality of evidence and called for more stringent studies with advanced imaging methods, comprehensive bone resorption metrics, longer follow-up periods, and larger sample sizes.^17,26^ Included in the reviews were 2 studies with CT imaging evaluating the mandible following BoNT/A masseter treatment for MMP. One 3-month study (*n* = 10) reported no bone changes.^27^ Another 6-month study (*n* = 20) reported bone volume decreases in participants treated twice with BoNT/A within 4 months (*n* = 10).^28^ Thus, the clinical evidence of mandibular bone changes following BoNT/A masseter treatment for MMP is limited.

The present analysis evaluated whether onabotA bilateral masseter treatment impacts mandibular bone density in healthy adults with MMP under a specific treatment paradigm. Multi-detector CT (MDCT) images collected in a prospective clinical study evaluating 1 or 2 onabotA treatments (24, 48, 72, or 96 U) over 1 year were examined.^29^ No clinically significant effects (ie, no signs of short- or long-term pathoses) of onabotA were observed at the assessed mandibular regions (condyle, premolar area, ramus) or bone types (cortical, trabecular). This study represents the most rigorous evaluation to date of mandibular bone density following onabotA MMP treatment.

## METHODS

### Study Design and Participant Selection

Analyses were performed on MDCT images collected from a 12-month, multicenter, double-blind, randomized, placebo-controlled, dose-escalation, Phase 2 study (NCT02010775) investigating safety and efficacy of onabotA treatment for MMP.^29^ Participants with investigator-rated bilateral marked (Grade 4) or very marked (Grade 5) MMP at baseline on the validated 5-grade Masseter Muscle Prominence Scale (MMPS) were treated with onabotA (24, 48, 72, and 96 U) or placebo. Participants were screened for enrollment in 1 of 4 cohorts consisting of ∼50 participants each under a pre-defined randomization schedule. Full details on participant enrollment and randomization are described in Carruthers et al.^29^ Participants who returned to bilateral marked or very marked MMP could be retreated on Day 180 with the same treatment received on Day 1. Study exit occurred at Day 360. Population exclusion criteria included history or evidence of TMD signs/symptoms, presence of metal implants interfering with CT imaging (determined by CT technologist), or clinically relevant abnormal findings hindering CT assessment (determined by maxillofacial radiologist).

MDCT scans were performed at baseline (pre-treatment), Day 90, and Day 360. The present analysis included MDCT images from participants who received onabotA ≥48 U or placebo and had images at all 3 time points (*n* = 123; placebo, *n* = 30; 48 U, *n* = 29; 72 U, *n* = 33; 96 U, *n* = 31). Participants missing MDCT scans at any time point or treated with onabotA 24 U were excluded, because onabotA 24 U showed limited efficacy for treating MMP.^29^

This study was conducted at 12 sites in Australia, Canada, and Taiwan between January 2014 and November 2017. The study was approved by local IRBs or independent ethics committees in accordance with the International Council of Hamonisation E6 guideline for Good Clinical Practice and the Declaration of Helsinki. All participants provided informed written consent.

### Treatment Administration

**Figure 1.**
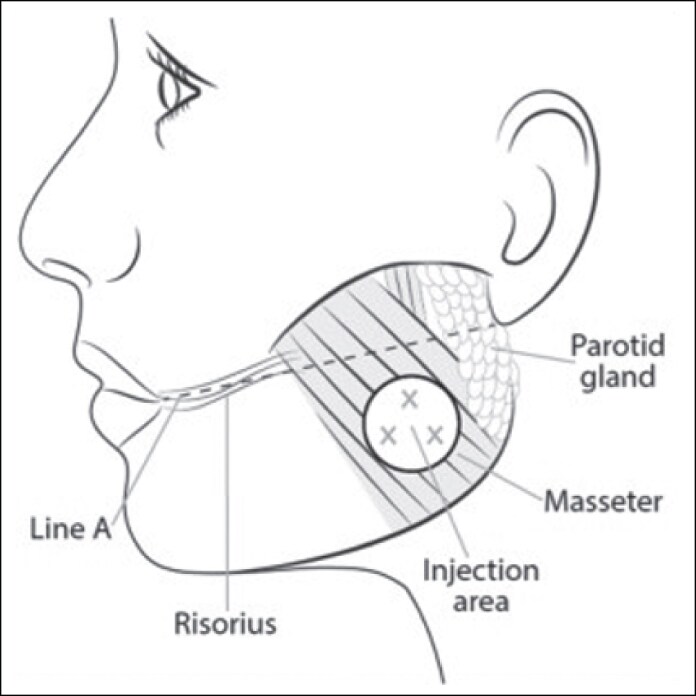
A diagram of the face depicting the injection area for the masseter.

On Day 1 of each treatment period, participants received onabotA or placebo as 6 total intramuscular injections (3 bilateral injections in a selected portion of the masseter; Figure 1). For each masseter muscle, the 3 injections were administered to the area of maximal muscle bulge, with each injection site ∼1 cm apart within the treatment area. The same treatment was readministered on Day 180 if participants met retreatment criteria. Treatment Cycles 1 and 2 comprised Days 1 to 180 and Days 180 to 360, respectively.

### Multi-detector CT (MDCT) Scan Methodology

Independent subject matter experts in nonclinical sciences, clinical dentistry, and maxillofacial radiology agreed with MDCT as an appropriate diagnostic modality to assess changes in mandibular bone density. Although dual X-ray absorptiometry (DXA) is employed for measuring bone mineral density (BMD) in vertebrae and long bones, it is not validated to assess craniofacial or maxillofacial bone density. DXA lacks the spatial resolution of CBCT and MDCT, both of which enable discrimination between cortical and trabecular compartments.^30^ For bone density assessments, MDCT imaging protocols with higher contrast-to-noise resolution are more reliable than CBCT.^31^

At each study site, each participant had low-dose MDCT scans taken pre-treatment at baseline and post-treatment at Days 90 and 360 (study exit) by a protocol-trained CT technologist with a calibrated CT device and exposure settings. Volumetric images were acquired with an in-plane resolution of 0.3 mm. A quality control process identified CT scans with artifacts or inadequate anatomic coverage to ensure their correct recapture. High-quality CT scans (bone kernel reconstructions) were assessed by 2 board-certified oral and maxillofacial radiologists.

### Assessments

#### Mandibular Regions of Interest

Mandibular bone density was assessed at defined locations in the condyle, premolar area, and ramus. These regions of interest (ROIs) were chosen due to their potential susceptibility to changes in biomechanical loading and bone density resulting from onabotulinumtoxinA-induced muscle weakness. Measurements at each location included the trabecular and cortical bone compartments separately or combined (integral density) (Table 1).

**Table 1.** Anatomic Locations Assessed Across the Mandible

Region	Landmark	Measure
Condylar head: integral bone density (trabecular + cortical HUs) Average Left Right	Medial and lateral poles of condyle define the central frontal plane Sections made in the central plane and 2-3 mm anterior and posterior to this plane	HU measurement within an ROI that includes condylar cortical and trabecular bone Determine average of triplicate measures in the anterior, central and posterior planes Separate and average measurements of the right and left condyles
Premolar/dentoalveolar: integral bone density (trabecular + cortical HUs) Right	1-mm-thick section parallel and immediately distal to the most anterior premolar	HU measurement within an ROI that includes both cortical and trabecular bone HU measurement within an ROI that includes trabecular bone only
Premolar/dentoalveolar: trabecular compartment bone density (HUs) Right		
Premolar/dentoalveolar inferior mandibular border: cortical bone density (HUs) Right		Point (pixel) HU measurement within the buccal, lingual and inferior cortex of the mandibular inferior border
Ramus: integral bone density (trabecular + cortical HUs) Right	The right and left lingula at the mandibular foramina define the coronal plane of section for measurements	HU measurement within an ROI that includes both cortical and trabecular bone HU measurement within an ROI that includes trabecular bone only
Ramus: trabecular compartment bone density (HUs) Right		
Ramus inferior mandibular border: cortical bone density (HUs) Right		Point (pixel) HU measurement within the buccal, lingual and inferior cortex of the mandibular inferior border

HU, Hounsfield units; ROI, region of interest.

The condylar head was selected in consideration of biomechanical loading exerted on this region during mastication and its susceptibility to clinical pathoses, including TMDs.^32,33^ The premolar area was selected as a dentoalveolar site with dynamic trabecular bone remodeling following edentulism.^34^ The ramus trabecular and cortical compartments were selected owing to their proximity to the masseter insertion site and potential susceptibility to the putative skeletal effects of masseter paresis. At each site, anatomic landmarks were applied to guide image generation for bone density measurements with standardized measurement locations for each CT scan.

#### Bone Density Measurement

Bone density was approximated through Hounsfield units (HUs), a relative quantitative measurement of radio density that correlates with BMD, and scaled according to the relative densities of air (−1024 HU) and water (0 HU).^35,36^ HUs are the appropriate measure for bone density on CT scans and are applied to evaluations of patients with suspected osteopenia or osteoporosis.^30,37^ Bone density changes over time were measured as the mean percent change from baseline in HUs for each time point at each anatomical location.

#### Bone Density Measurement Locations

**Figure 2.**
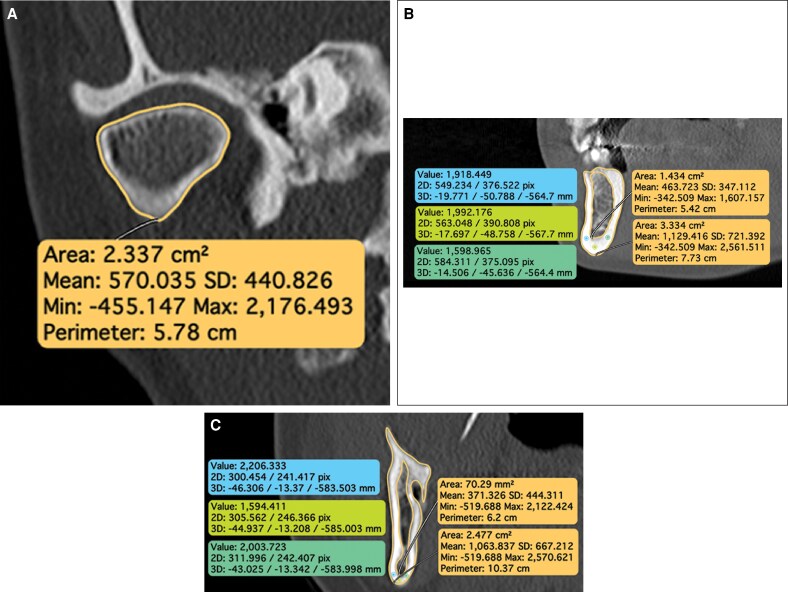
Example of (A) condyle, (B) premolar/alveolar, and (C) ramus measures. A single CT slice (1 or 3 mm, as appropriate) was employed for evaluations of all 3 regions of interest.

The density of the mandibular condyle was assessed bilaterally on 3-mm-thick slices, or 2-mm-thick slices when the anterior–posterior dimension was narrow (Figure 2A). Given its small size, low trabecular bone content, and thin cortical shell, the condyle was assessed as an integral measurement (ie, combined cortical and trabecular bone). To consistently and reliably segment the same anatomic extent of the mandibular condyle, the image volume was oriented along the long axes of each condyle in all 3 orthogonal planes. Three ROIs were analyzed to represent the posterior, central, and anterior regions of the condyle and encompass the full anterior–posterior dimension. Given the small volume of the condyles, measurements from these 3 regions were averaged to derive an integral bone density of each condyle.

For the premolar area, the image volume was oriented parallel to the long axes of the premolars, and 1-mm-thick slices were made through the interdental space, just distal to the right first premolar (Figure 2B). The ROI encompassed the entire circumference of cortical bone from the interdental alveolar crest to the inferior border and back. Trabecular bone density within this ROI was measured. Cortical bone density was measured at 3 distinct points within the cortex of the right inferior border of the mandible to include locations on the buccal, inferior, and lingual aspects of the cortical border. Each measurement was made at approximately the center of the total cortical width.

For the ramus, integral bone density was assessed at the site of masseter attachment on the right ascending ramus, near the region of the lingula at the mandibular foramen (Figure 2C). The image volume was reoriented with the occlusal plane parallel to the axial plane and the lingual of the right and left rami in the same coronal plane, and 3-mm-thick image slices were generated to measure trabecular and cortical bone densities. Trabecular bone density was analyzed within the endosteal region of the right ascending ramus. Cortical bone density was measured at 3 distinct points within the cortex of the right inferior border of the mandible to include locations on the buccal, inferior, and lingual aspects of the cortical border. Each measurement was made at approximately the center of the total cortical width.

#### Bone Density Measurement Reliability

Before the analyses, a single radiologist evaluated images from 4 participants, each 10 times. The bone density method reliability is reported as the median coefficient of variation (CV). The inter-rater reliability was evaluated by 2 radiologists, each evaluating images from the same 13 participants, and is reported through the intraclass correlation coefficient (ICC).^38^

### Statistics

All participants from the Phase 2 study who received onabotA (≥48 U) or placebo and had complete MDCT scans were included in the analysis. Changes in bone density at Day 90 and Day 360 were reported as mean percent change from baseline in HUs and presented as descriptive statistics with 95% CIs.

## RESULTS

### Participant Demographics and Study Disposition

Supplemental Figure 1 provides the study design schema and treatment arms. Of 187 participants enrolled and treated in the study, 167 (89.3%) completed the study (ie, received baseline treatment and had baseline, Day 90, and Day 360 MDCT scans), including 123 (65.8%) who received onabotA (≥48 U) or placebo (onabotA 48 U, *n* = 29; onabotA 72 U, *n* = 33; onabotA 96 U, *n* = 31; Placebo, *n* = 30). Primary efficacy and safety data are reported in Carruthers et al.^29^ Most participants were female (81.8%), of Asian descent (79.7%), with an average age of 35.4 years (range, 18-50 years). Baseline MMPS severity was primarily Grade 4 (78.1%) and similarly distributed across groups.

### Reliability of Bone Density Measures

The methodology to determine bone density in HUs was developed and verified by a board-certified oral and maxillofacial radiologist (S.M.M.). The median CV was calculated to assess the variability of repeated measurements by a single rater, with values ranging from 2.3% to 13.7%, indicating minimal-to-moderate variability (Table 2). Premolar trabecular bone and ramus inferior border (cortical bone) had the highest CVs, which was not unexpected due to the small size of these ROIs. Higher variability in trabecular vs cortical bone measurements was expected due to inherent variability in trabecular microarchitecture, including variable trabecular spacing and thickness.

**Table 2.** CV by Region and Bone Type

Anatomic region	CV (%)
Condyle, integral	3.9
Premolar, integral	2.3
Premolar, trabecular	12.5
Premolar, mandibular border (cortical bone)	3.6
Ramus, integral	2.4
Ramus, trabecular	5.7
Ramus, inferior border (cortical bone)	13.7

Standard deviation calculated as a percentage of the mean. CV, coefficient of variation.

The agreement between 2 independent raters was based on point estimates of ICC.^39^ Inter-rater reliability was high, with ICCs from 0.68 to 0.99 for all measures across all time points, indicating substantial to almost perfect agreement (Supplemental Table 1).

### Bone Density Analyses

**Figure 3.**
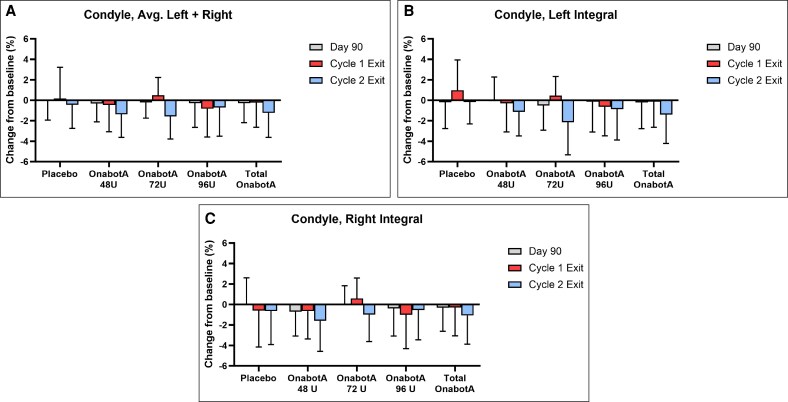
The mean percent change from baseline in integral (cortical + trabecular) bone density in HUs by dose group and visit for the (A) combined (average) left and right condyle, (B) left condyle, and (C) right condyle. Error bars indicate standard deviation. Cycle 1 Exit includes participants who received 1 treatment. Cycle 2 Exit includes participants who received 2 treatments. HU, Hounsfield unit; OnabotA, onabotulinumtoxinA.

**Figure 4.**
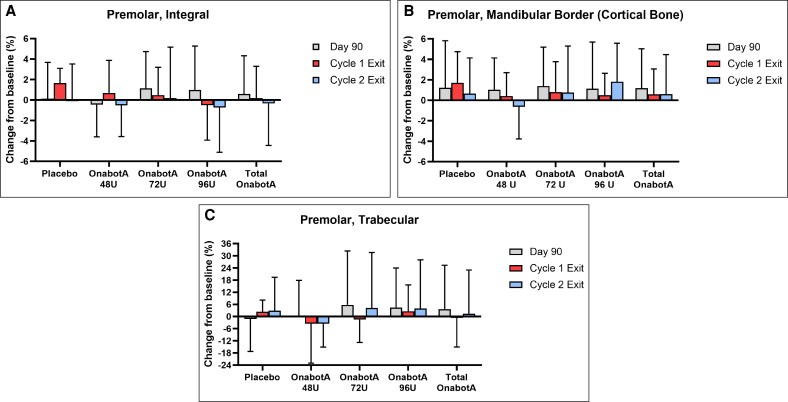
The mean percent change from baseline in (A) integral (cortical + trabecular) bone, (B) cortical bone, and (C) trabecular bone density in HUs by dose group and visit for the premolar area. Error bars indicate standard deviation. Cycle 1 Exit includes participants who received 1 treatment. Cycle 2 Exit includes participants who received 2 treatments. HU, Hounsfield unit; OnabotA, onabotulinumtoxinA.

**Figure 5.**
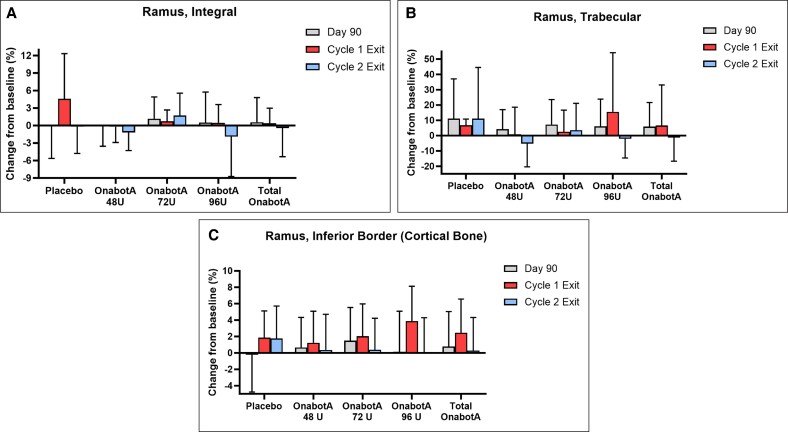
The mean percent change from baseline in (A) integral (cortical + trabecular) bone, (B) trabecular bone, and (C) trabecular bone density in HUs by dose group and visit for the ramus. Error bars indicate standard deviation. Cycle 1 Exit includes participants who received 1 treatment. Cycle 2 Exit includes participants who received 2 treatments. HU, Hounsfield unit; OnabotA, onabotulinumtoxinA.

Bone density changes for all ROIs were comparable between onabotA groups and placebo at each post-treatment time point, for 1 or 2 treatments. The mean percent change from baseline in bone density (HUs) for onabotA vs placebo is shown in Figures 3–5. The mean difference in percent change from baseline between onabotA and placebo is shown in Supplemental Tables 2-4.

#### Condyle

Baseline values for the integral assessment in the condyle were comparable among treatment groups for the left, right, and combined (average) of the left and right condyles (Supplemental Figure 2A). Overall, post-treatment HU ranges were similar across groups, with no apparent trends for treatment or time point. The greatest mean percent change in bone density in the condyle for any group across time points and treatments was a decrease of 2.15% (onabotA 72 U, Day 360, left condyle, 2 treatments) (Figure 3 and Supplemental Table 2). A decrease in bone density of 0.17% was observed at the same time point for placebo. Some increases in bone density were also observed; all were below 1%, with the greatest mean percent increase occurring in placebo (0.97%, left condyle, Day 360, 1 treatment). The greatest mean difference in percent change from baseline in the condyle (average, left, right) between onabotA and placebo across time points and treatments was a 1.98% decrease (onabotA 72 U, Day 360, left condyle, 2 treatments) (Supplemental Table 2).

Across all condyle measures, all but one 95% CI for the difference between onabotA doses and placebo included 0 (Supplemental Table 2), supporting similar change from baseline across time points and treatments despite sporadic instances of bone density decreases. Specifically, integral bone density was the only measurement where 95% CI did not include 0 (onabotA 72 U, Day 360, left condyle, 2 treatments), with a mean difference from placebo of −1.98% (95% CI, −3.69, −0.28). The results indicate that 1 or 2 treatments with onabotA 48, 72, or 96 U over 1 year do not significantly affect condyle bone density.

#### Premolar Area

Baseline values for the premolar ROIs were comparable among all onabotA groups and placebo for the integral, trabecular compartment, and premolar mandibular inferior border (Supplemental Figure 2B). The post-treatment HU range for each premolar measure for onabotA groups and placebo was similar for all time points and treatments. As expected from the small size and relatively low trabecular bone content of the ROI, premolar trabecular measurements had the most variable HU distribution (Table 2).

For premolar integral bone, the greatest mean percent change in density from baseline for any group across time points and treatment cycles was an increase of 1.65%, observed at Day 360 for participants treated once with placebo (Figure 4A and Supplemental Table 3). In comparison, bone density changes from baseline in the onabotA groups at the same time point ranged from a decrease of 0.74% (onabotA 96 U, 2 treatments) to an increase of 0.67% (onabotA 48 U, 1 treatment). Relative to placebo, the greatest percent decrease from baseline in premolar integral bone density across time points and treatment cycles was 2.15% at Day 360 for participants treated once with 96 U onabotA (Supplemental Table 3).

For premolar trabecular bone, the greatest mean bone density change from baseline for any group across time points and treatments was an increase of 5.75% (onabotA 72 U, Day 90) (Figure 4B and Supplemental Table 3). A decrease in bone density of 1.16% was observed at the same time point for placebo. Relative to placebo, the greatest percent decrease from baseline in premolar trabecular bone density across time points and treatments was 6.40% (onabotA 48 U, Day 360, 2 treatments) (Supplemental Table 3).

In the premolar inferior border (cortical bone), the greatest mean density change from baseline for any group across time points and treatments was an increase of 1.81% (onabotA 96 U, Day 360, 2 treatments) (Figure 4C and Supplemental Table 3). Only 1 instance of decreased premolar cortical bone density was observed (0.64%, onabotA 48 U, Day 360, 2 treatments), with all other time points and treatment groups exhibiting minor (<1.81%) density increases. Relative to placebo, the greatest percent decrease from baseline in premolar cortical bone density across time points and treatments was 1.30% (onabotA 48 U, Day 360, 2 treatments) (Supplemental Table 3). Together, these data indicate that 1 or 2 treatments with onabotA 48, 72, or 96 U over 1 year had negligible effects on premolar area bone density.

#### Ramus

Baseline values for the ramus ROIs were comparable among onabotA groups and placebo for the integral, trabecular, and inferior border of the ramus (Supplemental Figure 2C). Overall, the HU range for each ramus measure for onabotA and placebo was similar across time points and treatments. As expected, the ramus trabecular measure had the highest variability (Table 2).

For ramus integral bone, the greatest mean percent density change from baseline for any group across time points and treatments was an increase of 4.60% (Placebo, Day 360, 1 treatment). In comparison, bone density changes from baseline in the onabotA groups at the same time point ranged from a decrease of 1.89% (onabotA 96 U, 2 treatments) to an increase of 1.73% (onabotA 72 U, 2 treatments) (Figure 5A and Supplemental Table 4). Relative to placebo, the greatest percent decrease from baseline in ramus integral bone density across time points and treatment cycles was 4.81% (onabotA 48 U, Day 360, 2 treatments) (Supplemental Table 4).

For ramus trabecular bone, the greatest mean density change from baseline for any group across time points and treatments was an increase of 15.54% (onabotA 96 U, Day 360, 2 treatments) (Figure 5B and Supplemental Table 4). Similarly large bone density increases (11.07%) were also observed in placebo at the same time point. The increase in ramus trabecular bone density in the placebo group led to large relative decreases from baseline compared with onabotA groups, with the greatest relative decrease of 16.36% (onabotA 48 U, Day 360, 2 treatments) (Supplemental Table 4). However, all CIs for differences between onabotA groups and placebo included 0, indicating similar change from baseline between groups.

For ramus inferior border (cortical bone) measurements, the greatest mean percent change from baseline for any group across time points and treatments was an increase of 3.87% (onabotA 96 U, Day 360, 1 treatment) (Figure 5C and Supplemental Table 4). Only 1 instance of decreased ramus cortical bone density was observed (0.24%, placebo, Day 90), with all other time points and treatment groups exhibiting bone density increases. Relative to placebo, the greatest percent decrease from baseline in ramus cortical bone density across time points and treatments was 1.67% (onabotA 96 U, Day 360, 2 treatments) (Supplemental Table 4). These data indicate that 1 or 2 treatments with onabotA (≥48 U) over 1 year have minimal effects on bone density in the ramus.

## DISCUSSION

This analysis represents the first well-controlled, prospective evaluation of the potential effect of onabotA masseter treatment on mandibular bone density in a relatively large sample of healthy adults with MMP. The MDCT scans included in this study provided unprecedented quantitative insight into mandibular bone density, extending beyond the depth of analysis reported in the literature for any previous study. This analysis demonstrated that onabotA masseter treatment did not negatively impact mandibular bone density in a healthy adult MMP population. No clinically significant bone density decreases were observed when compared with baseline or placebo, across treatment groups (onabotA 48, 72, or 96 U), post-treatment time points (Day 90, Day 360), or number of treatments (1 or 2 over 1 year) in the 3 ROIs (ramus, premolar, condyle) and bone types (cortical, trabecular) evaluated.

MDCT scans were selected to assess mandibular bone density because they offer several advantages over other imaging modalities. MDCT scans provide a 3D view of the bone structure with high spatial resolution, allowing accurate discrimination between cortical and trabecular compartments.^30^ These attributes of MDCT enable quantification of changes within small ROIs, including subregions of the irregularly shaped mandible. MDCT scans provide volumetric information, and the protocol was designed to analyze complex regions for precise and direct assessments compared with areal BMD from 2D imaging modalities such as DXA.^21,40,41^ DXA cannot discriminate between cortical and trabecular compartments.^30^ Finally, although many studies have relied on CBCT or CT to assess mandibular bone changes after BoNT/A treatment of masticatory muscles, the lower resolution of these techniques might limit their ability to detect structural changes in fine regions of the mandible.^18-22,24-28^ MDCT scans were therefore considered appropriate to assess bone density, because they provide detailed and accurate information about both bone structure and its mineralization.

The rationale for assessing bone density at Day 90 and Day 360 was based on both the peak reduction in masseter muscle volume and recovery time, as described in the literature and confirmed in the Phase 2 study.^3,29,42,43^ Peak muscle volume reduction occurs around 90 days post-treatment, correlating with a reduction in bite force owing to muscle unloading, which could hypothetically lead to disuse-related bone changes within this period.^3,44^ Moreover, the ROIs were selected for their sensitivity to biomechanical loading and potential bone density variations induced by onabotulinumtoxinA-related muscle weakness. Maximum bite force reduction, and thus potential bone loss owing to adaptive response, manifests within 2 to 3 weeks post-treatment with BoNT/A in the masseter.^45^ Additionally, the Day 360 time point was chosen to capture potential long-term effects, such as safety concerns post-retreatment (1 or 2 treatments) or resolution of any changes observed at the 90-day time point. The timing of clinical assessments is consistent with findings from other human skeletal muscle disuse studies, which show rapid bone recovery upon resumption of skeletal loading.^46-48^ Thus, monitoring at these time points was considered adequate to capture any potential early adaptive bone loss as well as long-term bone density deficits.

Although changes in bone density relative to baseline were observed at multiple ROIs after onabotA, all were within the range of normal variations observed in the placebo group, consistent with no clinical significance. The greatest bone density decrease occurred in ramus trabecular bone, where a 5.29% decrease from baseline was observed at Day 360 in participants treated twice with onabotA 48 U. Ramus trabecular bone is relatively sparse compared with trabecular bone at the premolar and condyle regions, as evidenced by its low HU value at baseline. With lower baseline trabecular bone density, even minor absolute changes can result in large percent changes from baseline. Notably, the greatest density decrease in cortical bone in the ramus was <2% (Day 360, onabotA 96 U, 2 treatments), suggesting that the observed relative variability in the trabecular compartment might be partly owing to its inherently lower bone density. Furthermore, increases (>10%) in bone density in the ramus were observed in participants treated with onabotA 96 U and placebo. These increases and lack of predictable dose-related effects might reflect methodological difficulties in evaluating the sparse trabecular regions within the ramus compared with broader and relatively well-trabeculated regions such as the condyle and premolar areas. Caution should be exercised in interpreting trabecular bone changes in the ramus, where measurement variability could be confused with true biological effects.

The findings from this study contrast with previous preclinical reports of BoNT/A masseter treatments, which led to adaptive remodeling and reductions in mandibular bone density in models of skeletal disuse.^11,49,50^ Skeletal disuse studies rely on exaggerated reductions in muscle function to induce biomechanical unloading of the bone. There are also major differences in the anatomical and physiological properties of the masseter muscle and mandibular bone between humans and commonly studied laboratory animals. Mice and rats, for example, have much larger masseter muscles relative to the temporalis.^14,15^ This arrangement is opposite that of humans, suggesting selective paresis of the human masseter causes relatively less mandibular unloading compared with rodents.^51^ Rodents spend far more time chewing than humans, which may render rodents comparatively more vulnerable to mandibular bone loss during paresis of mouth closure muscles.^13,16,52^ These variations in experimental design and species-specific anatomical and physiological characteristics reinforce the difficulty of translating preclinical findings to humans.

In contrast, the present analysis focused on onabotA masseter treatment in healthy adults with MMP and found no clinically significant changes in mandibular bone density for up to 1 year post-treatment. Discrepancies between previous clinical reports and the present findings may be attributable to key differences in study designs and populations. Most studies reporting mandibular bone changes following BoNT/A injections focused on treating TMDs and myofascial pain with injections into both the masseter and temporalis muscles.^18-22^ Mandibular bone changes under these conditions may not be surprising, particularly in the mandibular condyle, where nearly 70% of the clinical TMD population exhibit resorptive or proliferative bone remodeling independent of treatment with BoNT/A.^53^ Injecting multiple mastication muscles in addition to the masseter may also lead to a greater reduction in mandibular loading and thus potential for bone remodeling. A retrospective analysis of a clinical TMD population found an inverse correlation between onabotA dose in the temporalis muscle and mandibular body trabecular bone density, supporting the notion that temporalis weakening may contribute to mandibular bone deficits in the setting of simultaneous masseter injection.^21^ These variables plausibly account for reports of decreased condylar cortical thickness and mandibular bone remodeling following BoNT/A masseter and temporalis treatment for TMDs and myofascial pain.^19,20,22^

Two previous studies yielded conflicting results on whether mandibular bone alterations occur in a healthy adult MMP population after BoNT/A masseter treatment. Consistent with the present findings, a retrospective review of CT data from 10 female participants with MMP found no changes in mandibular bone volume or thickness relative to baseline 3 months after a single treatment with BoNT/A in the masseters.^27^ A more recent, prospective, randomized trial employing CBCT imaging reported reduced bone volume at the mandibular gonial angle at Month 6 in 10 participants with MMP treated twice with BoNT/A within 4 months, but not in participants treated once.^28^ Though the reductions in bone volume at the mandibular gonial angle differed significantly between groups, no additional doses or areas of the mandible were analyzed, follow-up assessments were not performed to determine persistence of the effect, and safety consequences were not discussed. Moreover, 2 recent reviews challenged the findings reported in the literature and highlighted the need for more rigorous evaluations through specialized imaging techniques, measures of bone resorption, and larger sample sizes.^17,26^ Our analysis of MDCT scans at 7 mandibular sites in a large sample size found no clinically significant impact of onabotA (up to 96 U) on bone density in the condyle, premolar or ramus area with up to 2 treatments over 1 year. Therefore, our results support the conclusion that onabotA has no clinically significant (ie, no signs of short- or long-term pathoses) effect on mandibular bone density in healthy adults with MMP.

This analysis was based on prospectively collected MDCT scans from a randomized, placebo-controlled Phase 2 study. Given the overrepresentation of Asian participants, caution should be exercised in generalizing the findings to all populations. However, extensive research shows consistency in the mechanism of onabotA across ethnic groups.^54^ Moreover, inclusion of a disproportionate number of Asian participants was justified, because MMP treatment is prevalent in Asian cultures.^55^ Additionally, the study assessed changes as mean percent change from baseline in HUs at Day 90 and Day 360 based on MDCT under consistent acquisition settings. Although this HU approach does not provide direct information regarding absolute mineral density of bone matrix or bone tissue, this is not an obvious limitation for the current application, because no validated thresholds exist for absolute BMD values in any mandibular region.

## CONCLUSIONS

No negative impact on cortical or trabecular bone density in the condyle, premolar area, or ramus was observed in a healthy adult MMP population after 1 or 2 treatments with onabotA in the bilateral masseters under a standardized treatment paradigm.

## Supplemental Material

This article contains supplemental material located online at https://doi.org/10.1093/asj/sjaf167.

## Supplementary Material

sjaf167_Supplementary_Data
