# Supplementary material for: Longitudinal Computed Tomography Indicates No Negative Impact of OnabotulinumtoxinA on Mandibular Bone Density in a 12-Month, Double-Blind, Randomized, Repeat Treatment, Placebo-Controlled Study in Healthy Adults With Masseter Muscle Prominence
Source: Aesthet Surg J. 2025 Aug 22;46(1):76–85. doi: 10.1093/asj/sjaf167 (PMC12706864; doi:10.1093/asj/sjaf167)
Supplement: sjaf167_Supplementary_Data [file sjaf167_supplementary_data.zip › TableS1_ASJ-25-0485_BoneDensity.docx]

**Table S1.** Estimate of Degree of Agreement

| Estimate | Degree of Agreement |
| --- | --- |
| < 0 | Poor |
| 0 – 0.20 | Slight |
| 0.21 – 0.40 | Fair |
| 0.41 – 0.60 | Moderate |
| 0.61 – 0.80 | Substantial |
| 0.81 – 1.00 | Almost Perfect |
