# Supplementary material for: Longitudinal Computed Tomography Indicates No Negative Impact of OnabotulinumtoxinA on Mandibular Bone Density in a 12-Month, Double-Blind, Randomized, Repeat Treatment, Placebo-Controlled Study in Healthy Adults With Masseter Muscle Prominence
Source: Aesthet Surg J. 2025 Aug 22;46(1):76–85. doi: 10.1093/asj/sjaf167 (PMC12706864; doi:10.1093/asj/sjaf167)
Supplement: sjaf167_Supplementary_Data [file sjaf167_supplementary_data.zip › TableS2_Kostenuik_BoneDensity.docx]

**Table S2.** Baseline and Percent Change From Baseline in Bone Density in HUs in the Condyle

onabotA, onabotulinumtoxinA
Cycle 1 Exit includes participants who received 1 treatment; Cycle 2 Exit includes participants who received 2 treatments. Both exit visits are Day 360.
Combined (integral cortical bone + trabecular bone) bone density: Outlines the periosteal surface and encompasses all bone tissue within this perimeter.
Trabecular bone density: Outlines the endosteal surface and encompasses all bone tissue within this perimeter.
Inferior border bone density: Three discrete point measurements within the cortex at the inferior border of the mandible.
