# Supplementary figures and images for: Longitudinal Computed Tomography Indicates No Negative Impact of OnabotulinumtoxinA on Mandibular Bone Density in a 12-Month, Double-Blind, Randomized, Repeat Treatment, Placebo-Controlled Study in Healthy Adults With Masseter Muscle Prominence
Source: Aesthet Surg J. 2025 Aug 22;46(1):76–85. doi: 10.1093/asj/sjaf167 (PMC12706864; doi:10.1093/asj/sjaf167)

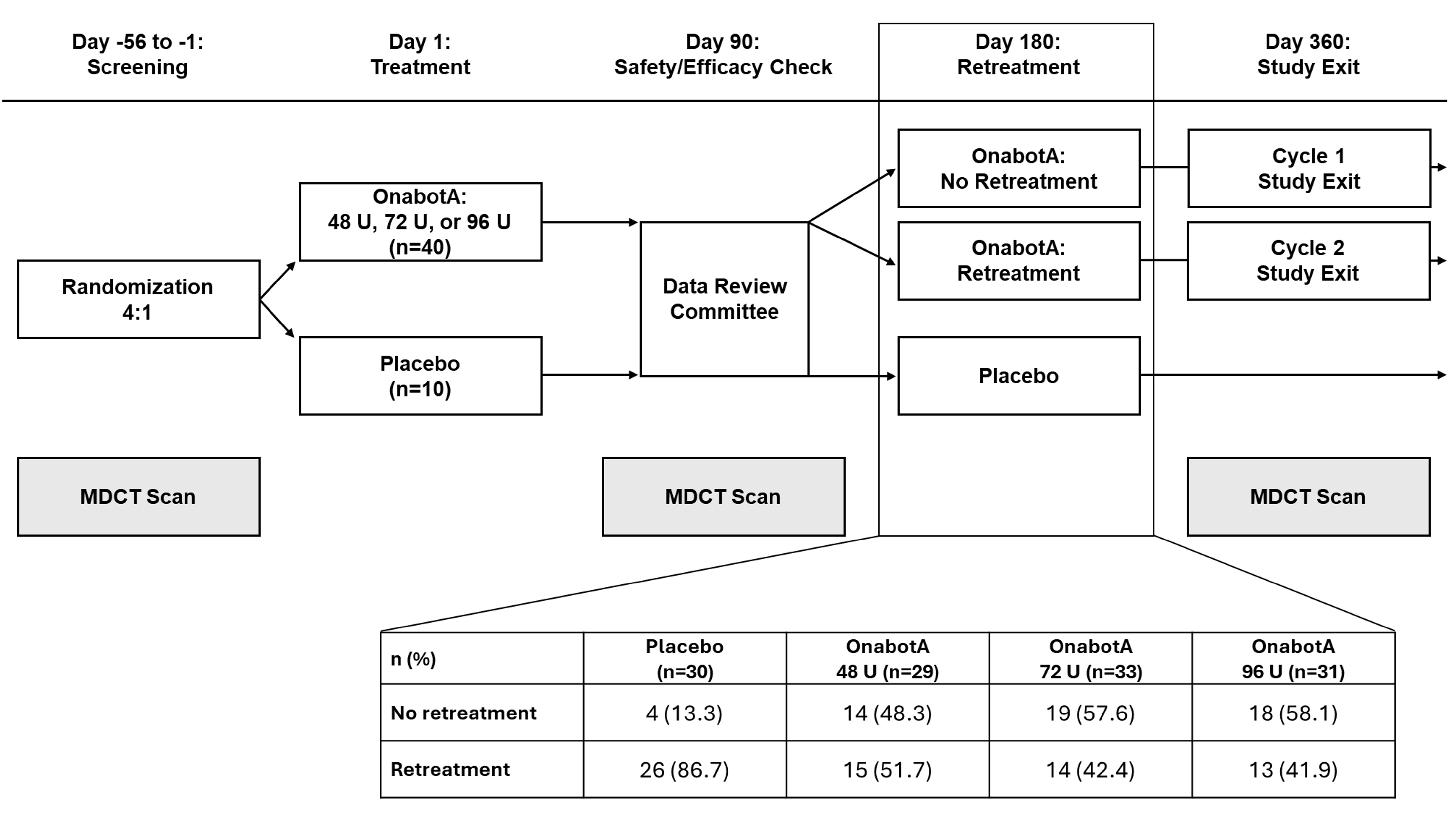

Supplement: sjaf167_Supplementary_Data [file sjaf167_supplementary_data.zip › FigureS1_ASJ-25-0485_BoneDensity.tif]

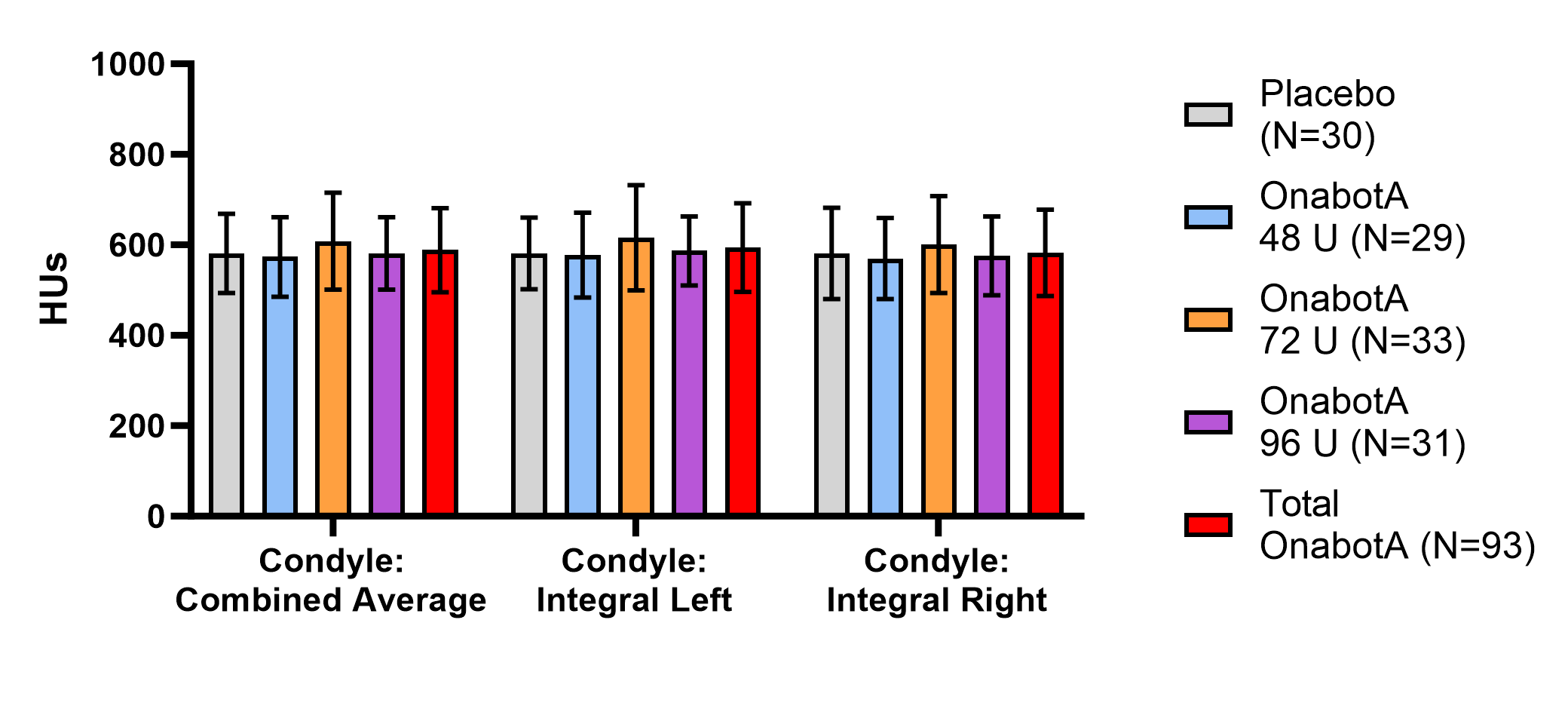

Supplement: sjaf167_Supplementary_Data [file sjaf167_supplementary_data.zip › FigureS2A_ASJ-25-0485_BoneDensity.tif]

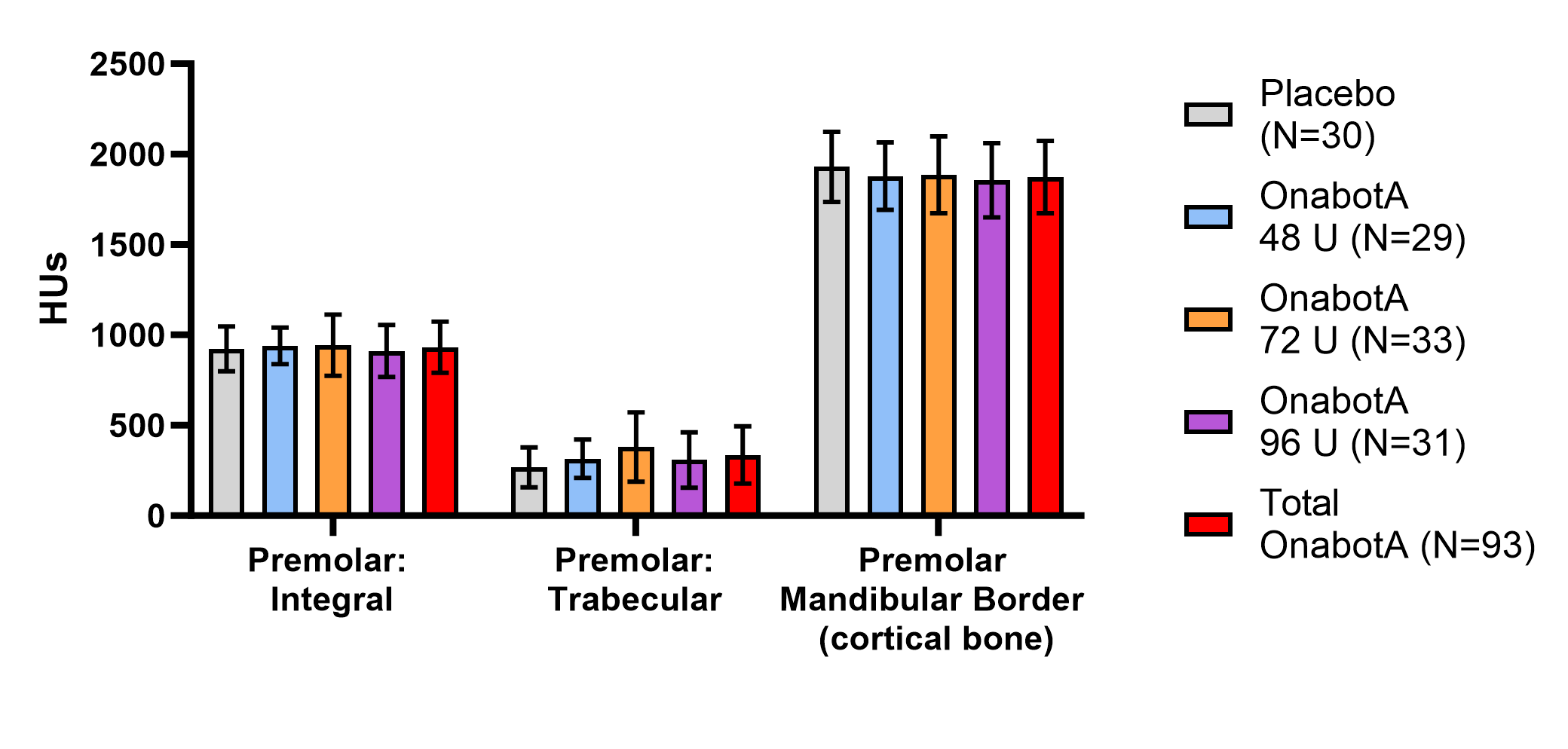

Supplement: sjaf167_Supplementary_Data [file sjaf167_supplementary_data.zip › FigureS2B_ASJ-25-0485_BoneDensity.tif]

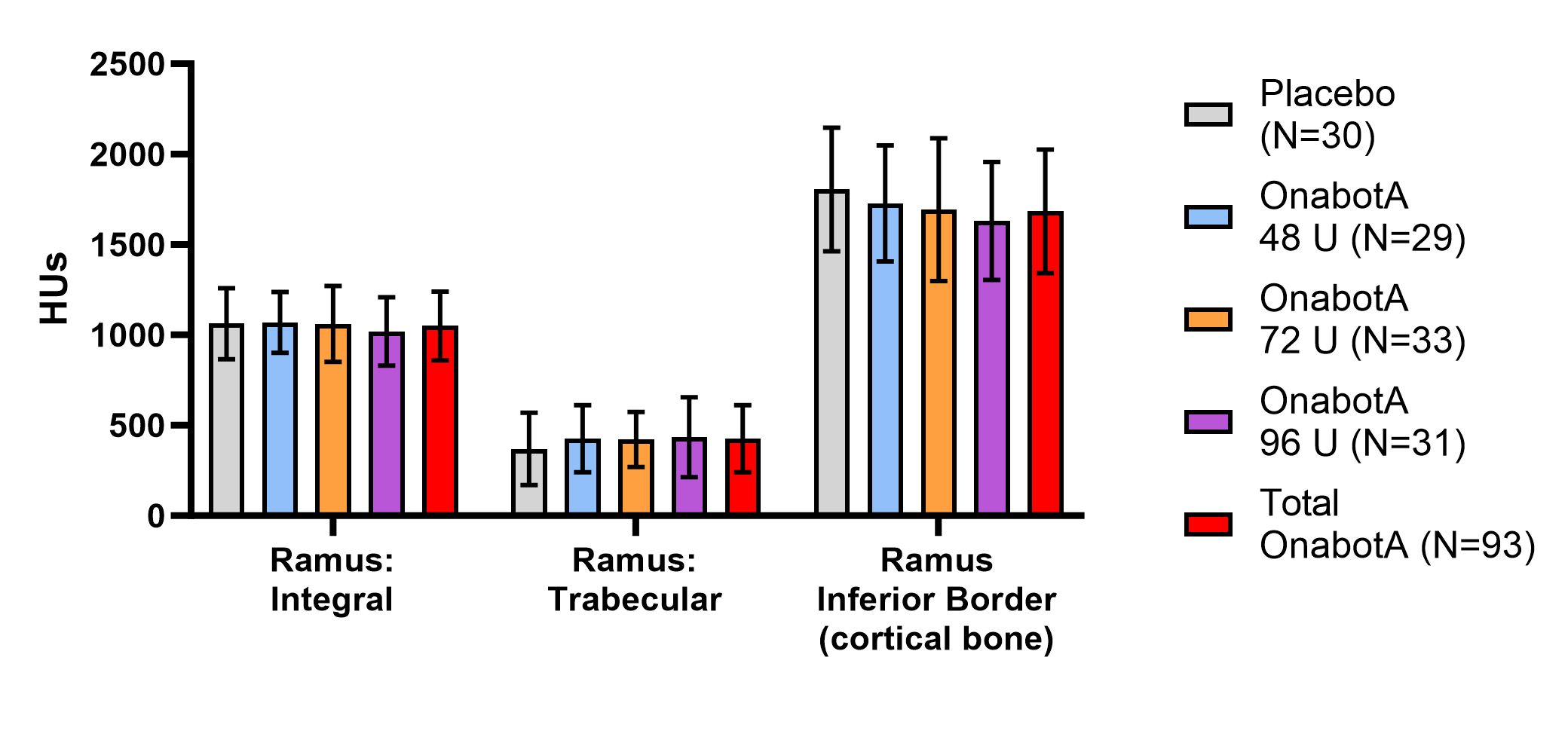

Supplement: sjaf167_Supplementary_Data [file sjaf167_supplementary_data.zip › FigureS2C_ASJ-25-0485_BoneDensity.tif]
